# A retrospective study on dystocia in the cat, evaluation of 111 cases

**DOI:** 10.1186/s13028-025-00805-w

**Published:** 2025-04-12

**Authors:** Eva Axnér, Josefine Jakobsson, Tilde Vermelin, Ulrika Hermansson

**Affiliations:** 1 Department of Clinical Sciences, Swedish University of Agricultural Sciences, P.O. Box 7054, Uppsala, SE 750 07 Sweden; 2 Small Animal Clinic, Swedish University of Agricultural Sciences, P.O. Box 7040, Uppsala, SE 750 07 Sweden

**Keywords:** Feline, Kitten, Obstetrics, Parturition, Reproduction

## Abstract

Dystocia affects on average 3–8% of all pregnancies in purebred cats. Nonpedigree cats are also affected, but the incidence is unknown. The causes of dystocia and the optimal treatment are largely unexplored in cats. The aims of the present retrospective study were to describe feline dystocia cases and to evaluate kitten mortality in relation to factors associated with dystocia in cats. Medical records of 111 cases (107 queens) treated for dystocia from 2017 to 2024 were retrieved from client files at the University Animal Hospital in Uppsala, Sweden. At the initiation of treatment, 276 kittens remained *in utero* or in the birth canal. The total kitten mortality rate, including that of kittens born before treatment but excluding four kittens that were euthanized at the owner’s request, was 40.9%. The mortality of kittens born after treatment was 44.1%, excluding four kittens euthanized at the owner’s request. Two queens died, one of which was euthanized at the owner’s request. Among all the cases, 91 (82.0%) were surgically treated, with caesarean section, or en bloc resection in two patients. Ovariohysterectomy was performed in 47.2% of the queens that were surgically treated. Medical treatment was initiated in 30 patients, and was successful in 11 of them, and 19 were further surgically treated after only partial or no success. The success rate of medical treatment was thus 36.7%. Eight queens were hypocalcaemic. Maternal hyperglycaemia was present in 65.5% of the cases and significantly increased the risk of kitten mortality. The estimated duration of second-stage labour before admission did not affect kitten mortality. Disturbed labour (total or partial uterine inertia) was the most common cause of dystocia. Feline dystocia was associated with high kitten mortality but low mortality in queens. Most queens with dystocia were treated surgically, but medical treatment with calcium and/or oxytocin was efficient in cases with non-obstructive dystocia, and ≤ 3 foetuses remaining. Hypocalcaemia may contribute to dystocia in a minority of cases. Maternal hyperglycaemia increased the risk of mortality before discharge. Diagnosing dystocia may be challenging in cats, as there is no clear association between the length of the parturition process and mortality.

## Background

Dystocia affects an average of 3–8% of all pregnancies in purebred cats [1–4] and varies with breed [5]. The frequency of dystocia in nonpedigree cats is more difficult to estimate, as the proportion that is bred generally is unknown [5]. Irrespective of breed group, dystocia affects the well-being of the pregnant queen and is associated with increased kitten mortality compared with natural parturition [2, 6]. The causes of dystocia and the optimal treatment options are largely unexplored in this species. Guidelines for the definition and treatment of dystocia in cats are therefore often extrapolated from recommendations for dogs. Apart from the absence of guidelines, diagnosing dystocia in cats may be more challenging than in canines. While an increased interval between the birth of puppies is associated with increased puppy mortality, no such association has been found in cats [7–9]. Although the total parturition length is usually < 6 h (h) and > 90% of all kittens are born within 2 h of each other, there is a very large variation in parturition length in cats [3, 8, 10]. Live kittens are often reported despite an interval exceeding 40 h [3, 11]. In addition, many cat pregnancies are unplanned [12], making it difficult to calculate the expected due date, as the date of breeding may not be known. The largest medical record study on feline dystocia included 158 cats but did not evaluate data on outcomes for the kittens [13]. There is, thus, a paucity of data on the effects of different treatment options and case characteristics on kitten mortality, with recent studies including no more than 48 cases [6, 14–16]. Evaluating data on feline dystocia cases would contribute to increased knowledge about how to handle these situations and might identify new areas of interest for future research on normal and abnormal feline parturition. The aim of the present retrospective study was, therefore, to describe feline dystocia cases, including haematology and serum biochemistry, and the results of diagnostic imaging. Furthermore, we aimed to compare the effects of different treatments and evaluate kitten mortality in relation to factors associated with dystocia in cats.

## Methods

### Medical records

The digital medical records of cats treated for dystocia between 1 Jan 2017 and 22 Apr 2024 were retrieved from the medical records system at the University Animal Hospital (UDS, Swedish University of Agricultural Sciences). The main inclusion criterion was that the queen should have been treated for dystocia at the animal hospital. Patients discharged because the queen was not pregnant or sent home because it was not considered dystocia were not included. Cats that were sent to other clinics for treatment because of restricted opening hours in the years 2023–2024 were also not included. Data on age, breed, pregnancy length, number of kittens born before and after the initiation of treatment, and the time of first signs of second-stage labour and treatment were collected. When available, data from a previous caesarean section (CS) were also collected. If the pregnancy length consisted of interval range because of multiple days of mating, the longest possible interval was used for consistency. The duration of time in second stage labour before examination was recorded as the time between the first observed sign of the second stage, i.e., abdominal contractions or the passage of foetal fluids, and the registered time of admission. The first sign of second-stage labour was not always clear and was sometimes recorded as the birth of the first kitten in the litter. Therefore, the interval used in this study was the minimum possible interval. Weak kittens that were euthanized because of poor response to resuscitation attempts were included in the kittens that died before discharge.

The results of haematology and blood chemistry (haematocrit (HCT), ionized calcium, and serum glucose), ultrasound examination, and radiography were also collected and related to outcomes.

Observed signs of dystocia were evaluated and categorised. Dystocia was classified into different categories from the information in the medical records. Uterine inertia was divided into total (i.e., no kittens born before initiation of treatment) or partial (i.e., kittens born before initiation of treatment) inertia [13]. Malformations and dead foetuses were recorded as foetal causes if they were considered to have contributed to dystocia. In several cases, it was not possible to attribute a specific cause.

Ethical permission was not needed because of the retrospective nature of the study.

### Statistics

Statistical calculations were performed in R (R Core Team, 2022) [17] and Minitab 21.4.1 (© 2023 Minitab, LLC, https://www.minitab.com). Pedigree and domestic queens were compared with the Mann‒Whitney test for age, parity, litter size, time in the second stage before admission, and time between admission and CS. Fisher’s exact test was used to compare the proportion of queens with known pregnancy lengths between pedigree queens and nonpedigree queens. It was also used to compare frequency of ovariohysterectomy (OHE) between queens that had undergone a previous CS and those that had not.

Time between admission and CS for queens that were medically treated first, were compared with queens that were subjected to CS without previous medical treatment with a two-sample t test after checking the normal distribution with Ryan–Joiner’s test.

To model the outcome, kitten death before discharge, a binomial generalized mixed model was used (glmer, family binomial, Control, optimizer = “bobyqa”). Four different binomial models were used: (1) hypoglycaemia and anaemia as binomial factors; (2) ultrasound diagnosis of foetal stress as a categorical factor; (3) time in the second stage as an ordinal categorical factor; and (4) treatment as a categorical factor. Litter was included as a random factor in all the binomial regression models. Significant results were further evaluated pairwise with Fisher’s exact test.

P values < 0.05 were considered significant. The results are shown as median and IQR (interquartile range) or as mean ± SD unless stated otherwise.

## Results

**Fig. 1:**
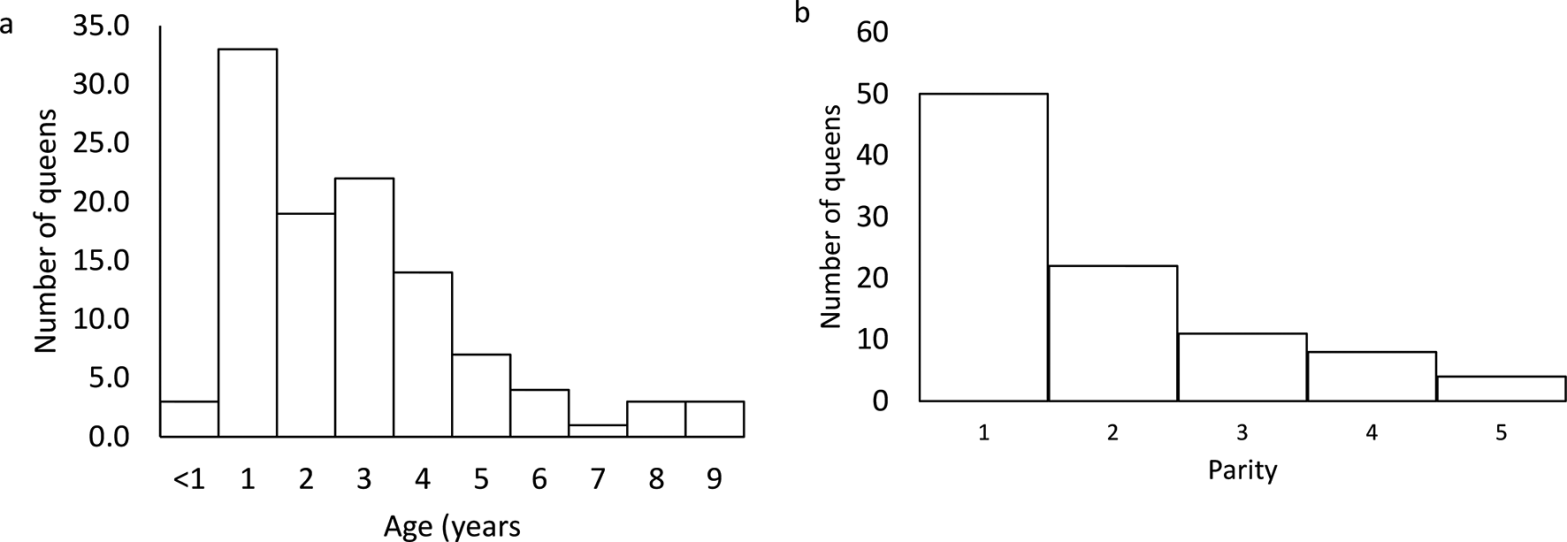
Distribution of ages and parities **a**) Distribution of ages of the queens **b**) Distribution of parity

**Fig. 2:**
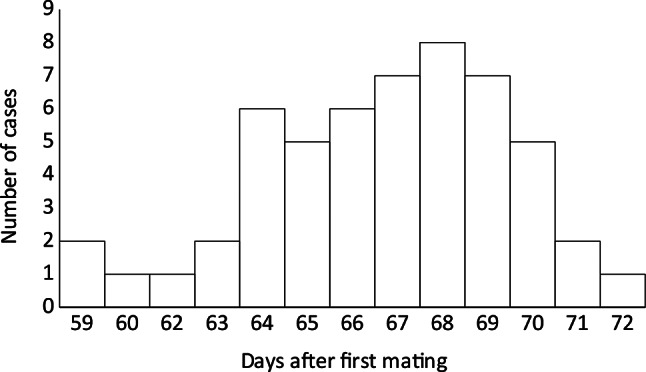
Days after first mating in 53 cases with known data

A total of 111 medical records fulfilled the inclusion criteria. Four queens were treated in two different pregnancies; thus, 107 individual queens were included. Seventeen breeds/breed groups were represented (Table 1). Ages varied between 9 months and 9 years (median 3.0, IQR 2.5, Fig. 1a) in 109 known cases, and parity varied between 1 and 5 (median 1.0, IQR 1.00, Fig. 1b) in 95 known cases. The number of days after the first mating varied between 59 and 72 days (median 67.0, IQR 4.0; Fig. 2). A significantly greater proportion of pedigree queens than Domestic Short- or Longhair queens had known data about the estimated day of pregnancy since mating (44/69 vs. 9/42, *P* < 0.001). There were no significant differences between pedigree- and domestic queens in age, parity, litter size, time in the second stage before admission, or time between admission and CS (*P* > 0.05). In 12 of 42 multiparous cases with known data, the queen had undergone a previous CS.

**Table 1:** Distribution of breeds in the study

Breed	Cases	Individual queens
Domestic Short- or Longhair	42	42
British Short- or Longhair	11	11
Ragdoll	11	11
Birman	7	7
Norwegian Forest cat	6	6
Bengal	5	3
Siberian	5	5
Persian/Exotic	4	4
Maine Coon	3	2
Neva Masquerade	3	3
Devon Rex	3	3
Abyssinian	3	3
Ocicat	2	2
Oriental	2	2
Turkish Angora	2	1
LaPerm	1	1
Cornish Rex	1	1

### Signs of dystocia

In 94 cases, the queen had one or more of the following signs: the queen being tired or in distress, prolonged or ceased contractions, expulsion of foetal fluids > 4 h previously, abnormal vaginal discharge, day 72 without birth, or a foetus that was visibly stuck in the birth canal (Table 2). In 12 cases without any of these signs, diagnostic imaging revealed suspected foetal malpresentation (*n* = 3), foetal stress (*n* = 3), foetal death (*n* = 5), or suspected mummified foetus (*n* = 1) as signs of suspected abnormal parturition. One queen had given birth to four kittens four days previously and had three viable kittens still in the uterus. One remaining queen had no obvious signs of abnormal parturition (24 h since the birth of the last kitten and no signs of foetal stress or foetal death). Although an interval of > 24 h between the birth of live kittens might be considered normal, this queen was included, as she was treated for dystocia with oxytocin and CS.

**Table 2:** Signs of dystocia

Sign of dystocia^#^	Number of cases
Prolonged contractions or expulsion of foetal fluids > 4 h^‡^	53
Abnormal vaginal discharge^†^	30
Queen described as tired or distressed	22
Partial expulsion	13
Foetus palpable in birth canal	6
Day 72 without birth, foetal HR decreased since previous day	1
**None of the above**,** miscellaneous**	
Foetal stress on ultrasound	3
Malpresentation suspected on x-ray	3
Last kitten born 24 h ago, normal foetal HR*	2
Last kitten born 15–20 h ago, FD	2
Last kittens born 3 days ago	1
No signs of parturition day 70, FD	1
One stillborn kitten at home 2 days ago, FD	1
Two stillborn kittens at home, 5 h ago, FD	1

^**#**^Queens could have more than one of these clinical sign, ^‡^ strong contractions > 30 min or weak > 4 h, ^†^purulent, discoloured, foul or haemorrhagic discharge, *one of these had given birth to a stillborn kitten and had a suspected mummified foetus on x-ray. FD = foetal death diagnosed on ultrasound

### Litter size and mortality

**Fig. 3:**
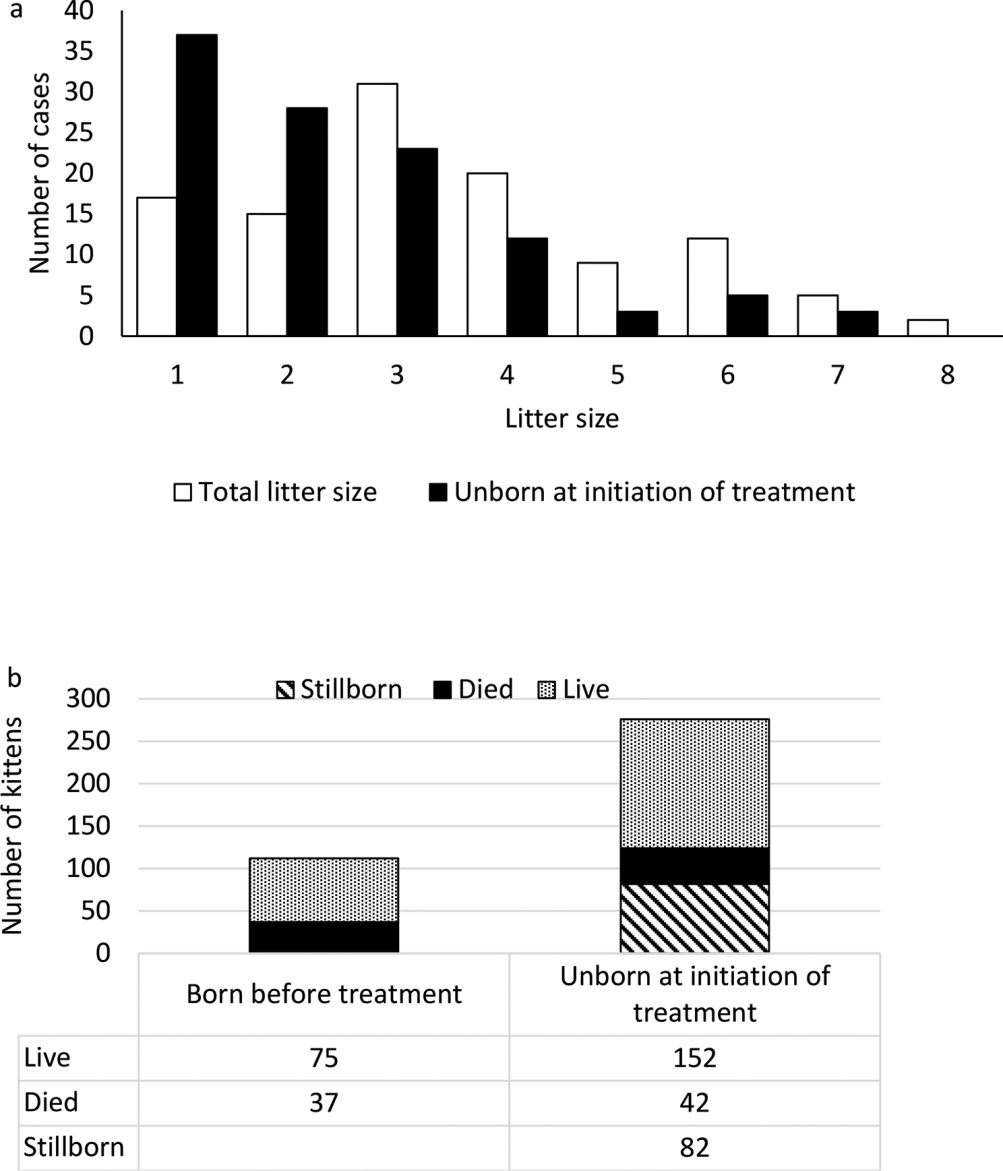
Kitten mortality. **a**) Distribution of total litter sizes and kittens still in utero or in the birth canal at initiation of treatment. **b**) Total number of kittens born before and after treatment, stillborn and live-born that died before discharge (including four kittens that were euthanized at the owner’s request). Live-born that died before admissions are included in “Died” for kittens born before treatment, as it was not always clear when a kitten died at home

The included queens gave birth to 388 kittens with a median litter size of 3.0 (IQR 3.0, range 1–8; Fig. 3a). In 51 litters, 112 kittens were born before the initiation of treatment. Thus, 276 kittens remained *in utero* or in the birth canal at the initiation of treatment. Seventeen (15.3%) litters comprised a single kitten (Fig. 3a). Among the kittens born after treatment, 44.9% died before being discharged. Excluding four kittens euthanized at the owner’s request, 44.1% died before being discharged (Fig. 3b). The total kitten mortality rate, including that of kittens born before treatment but excluding four kittens that were euthanized at the owner’s request, was 40.9%. Two of the queens included in the study died, one during surgery and one was euthanized during labour at the owner’s request because of financial concerns.

### Time in the second stage in relation to kitten mortality

**Fig. 4:**
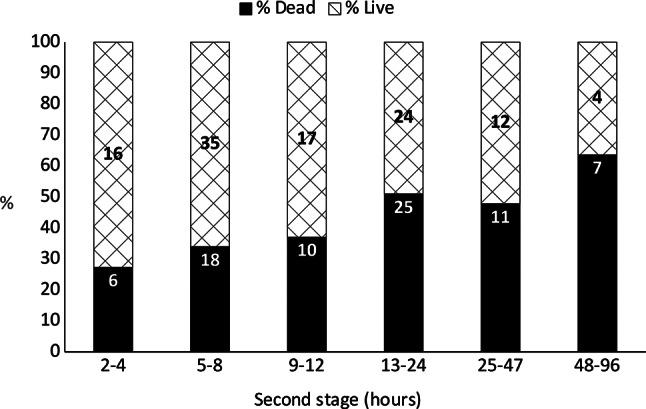
Kitten mortality before discharge at different intervals between start of second stage and discharge. Numbers of kittens displayed within bars. *P*>0.05

There was no significant effect of increased time in the second stage before admission on kitten mortality (*P* > 0.05, Fig. 4). Although kitten mortality was high in the six cases that were estimated to have been in the second stage at ≥ 48 h, four viable kittens were born after this time. One Ragdoll queen had three viable kittens delivered by CS three days after the birth of four kittens at home.

### Treatment and blood parameters

Among all 111 cases, 91 (82.0%) were surgically treated by CS, or OHE en bloc in two patients. In 43 cases, OHE was performed at surgery (including OHE en bloc). The frequency of OHE was significantly greater for queens that had undergone a previous CS (10/12 vs. 24/81 that had not undergone previous CS, *P* = 0.001). Nine queens that underwent OHE did not have known data about previous CS. In eight cases, the OHE was performed because of the condition of the uterus at CS. The other OHEs were performed, either at the owners’ request (*n* = 27) or at the recommendation of the veterinarian for other causes, such as not to continue breeding from a queen with problematic parturition (*n* = 5). In three cases, it was not obvious from the medical record why the OHE was performed. Medical treatment was initiated in 30 cases, and was successful in 11 of them, and 19 were further surgically treated after only partial or no success (Tables 3 and 4). The success rate of medical treatment was thus 36.7%. The time between admission and the initiation of surgery was significantly greater for queens that were first medically treated than for queens that were subjected to CS without previous medical treatment (313.5 ± 171.4 vs. 166. 8 ± 81.05 min, *P* < 0.001). Kitten mortality was significantly lower in cases in which CS was performed after previous medical treatment, compared with CS without previous attempts of medical treatment (Table 3).

**Table 3:** Outcome of different treatments

	Number of cases (%)	Unborn kittens Median (IQR) range	% dead before discharge of kittens born after treatment (proportion)
Digital manipulation only	8 (7.21)	1.0 (0.8) 1–3	63.6%^a^ (7/11)
Medical treatment only	11 (9.9)	1.0 (2.0) 1–3	31.6%^ab^ (6/19)
Medical treatment and CS	18 (16.2)	2.00 (2.2) 1–6	28.6%^b^ (14/49)
CS without previous medical treatment	71 (63.6)	2.0 (3.0) 1–7	47.6%^a^ (91/191)
Medical treatment and OHE en bloc*	1 (0.9)	1	1/1
OHE en bloc only*	1 (0.9)	4	4/4 kittens were euthanized at the owners request
Euthanasia*	1 (0.9)	≥ 1	≥ 1 (a stuck kitten was already dead)

^abc^Different letters within column = significant difference. *Descriptive data only

**Table 4:** Results of medical treatment

	Number of cases	Complete success (% success)	No effect	Partial success
Calcium only	12	4 (33.3)	7	1
Calcium and oxytocin	7	4 (57.1)	3	0
Oxytocin only	11	3 (27.3)	7	1

Medical treatment was only successful in queens with ≤ 3 kittens remaining unborn at the initiation of treatment. Oxytocin was administered efficiently at doses ranging from 0.03 IU to 0.1 IU, intravenously (*n* = 13), intramuscularly (*n* = 3) or subcutaneously (*n* = 1). Information about the route of oxytocin administration was not available in one case. Five queens were given a second dose of oxytocin, whereas no queen was given ≥ 3 doses. Calcium chloride dihydrate was diluted in saline as an intravenous infusion at doses of 0.2–2.5 mL/cat (0.05–0.5 mL /kg, Kalcium APL, 18 mg/mL, 0.45 mmol/mL; Apotek Produktion & Laboratorier AB).

Eight queens were hypocalcaemic (Ca^2+^ <1.20 nmol/L, Table 5), three of which were treated with calcium IV, the other five were treated surgically without previous medication. Only one of these patients, which also received one dose of 0.05 IU oxytocin IV, gave birth to all remaining kittens. Four queens without hypocalcaemia gave birth to all remaining kittens after treatment with calcium only (1–3 kittens). However, they were in the lower range of normocalcaemia (1.21–1.22 nmol/L).

**Table 5:** Results of serum analyses

Parameter Reference range	Ionised calcium 1.20–1.40 mmol/L	Glucose 3.9–6.7 mmol/L	Hematocrit (HCT) 29–55%
Below lower reference value	8.9% (8/90)	0% (0/87)	21.8% (19/87)
Within reference range	84.4% (76/90)	34.5% (30/87)	78.2% (68/87)
Above reference range	6.7% (6/90)	65.5% (57/87)	0% (0/87)

None of the queens was hypoglycaemic, whereas 65.5% of those with known values were hyperglycaemic (Table 5). Hyperglycaemia in the queen was associated with a higher mortality rate in kittens before discharge (*P* = 0.0041). Among queens with known values, 21.8% were anaemic (Table 5). Maternal anaemia was not associated with a higher kitten mortality (*P* = 0.54). The queen with the lowest HCT (19.3%) died, however, during surgery. A queen with a HCT of 22.6% recovered after receiving a xenotransfusion (canine blood) because of profuse vaginal bleeding, poor general condition and pale mucous membranes. At surgery, a partial uterine rupture was found.

### Ultrasound and radiography

**Fig. 5:**
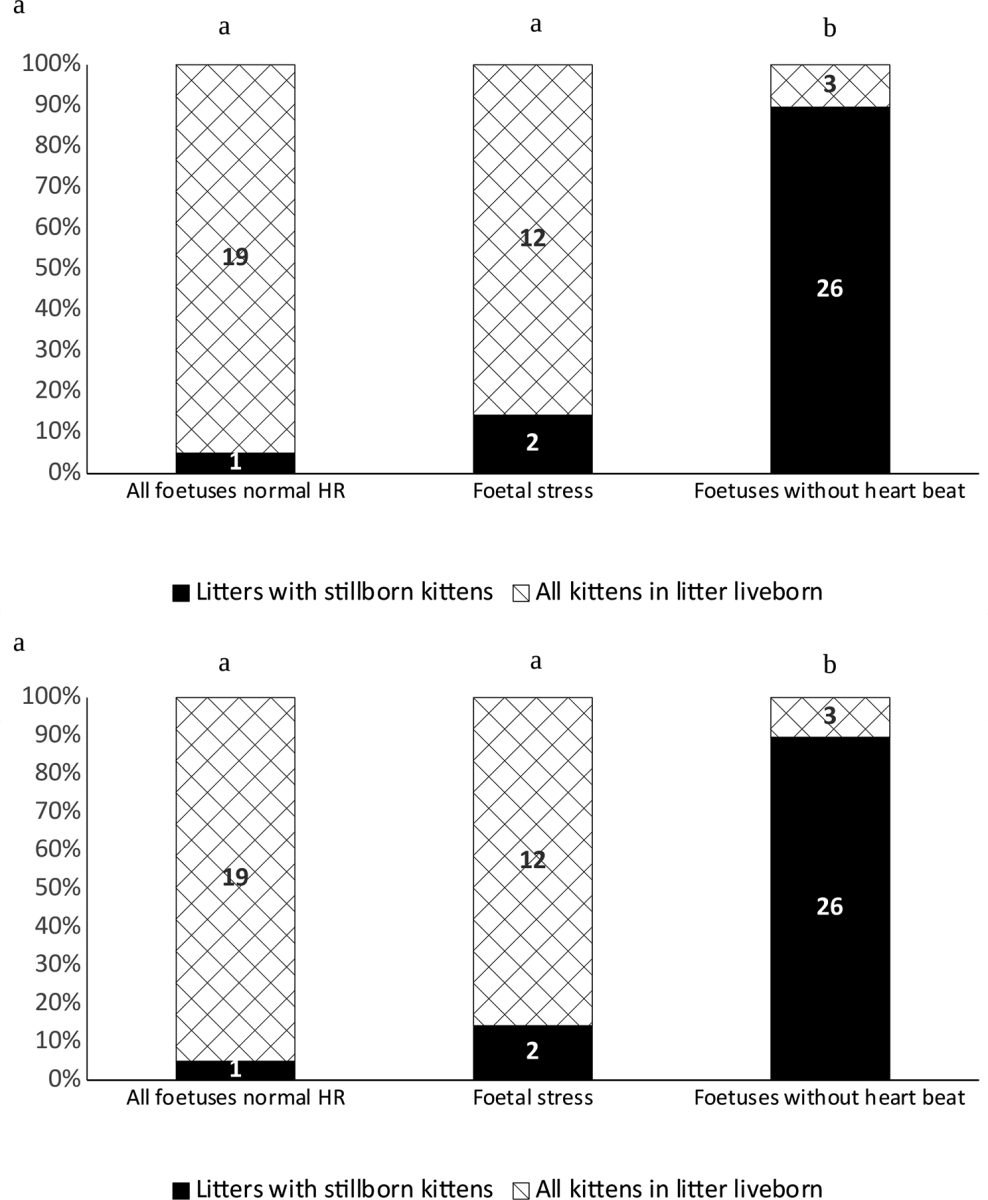
Kitten mortality in relation to ultrasound diagnosis. All fetuses normal=all observed fetuses had a heart rate ≥180 bpm. Fetal stress=one or more fetuses having a low heart rate, but no dead fetuses observed, Fetuses without a heartbeat=one or more fetuses had no detectable heartbeat. **a**) Litters with stillborn kittens after ultrasound evaluation in relation to ultrasound diagnosis of fetal heath. Numbers of litters displayed within bars. **b**) Mortality of individual kittens in the different categories. Numbers of kittens displayed within bars. ^ab^Bars with different letters differs

Diagnostic imaging was performed, after admission, for 99 of the cases. In 35 cases, only radiography was performed; in 16 cases, only ultrasound was performed, and in 48 cases, both ultrasound and radiography were performed. Litters with one or more foetuses without a detectable heartbeat at ultrasound evaluation had a greater risk of having one or more dead kittens and a greater total proportion of the kittens were dead or died, confirming the accuracy of the diagnosis (Fig. 5a, b). However, in three litters, all the unborn kittens were alive at discharge, although foetuses without heartbeats were detected at ultrasound. There was no significantly increased risk of having one or more dead kittens in litters with one or more foetuses showing a low heart rate as a sign of foetal stress, but no foetuses without a heartbeat. In 59 cases, an estimation of the number of dead foetuses was recorded. The number of dead foetuses was overestimated at ultrasound in five cases, in which nine kittens were live-born although they were not diagnosed with a heartbeat. In six cases, the number of stillborn kittens exceeded the number of foetuses diagnosed as dead at ultrasound. Twenty-nine foetuses did not have a detectable hearbeat on ultrasound evaluation. In these litters, a total of 36 kittens were stillborn. The number of kittens counted at radiography was correct in 76/77 cases. In the case with an incorrect estimation, 7 kittens were unborn, but 6 had been counted at radiography.

### Causes of dystocia

More than half of the dystocias were considered to have a maternal cause, of which 46.8% were partial or complete uterine inertia (Table 6). One queen was considered to have iatrogenic inertia because she had been given medroxyprogesterone acetate pills for oestrus suppression until 2 weeks before admission as the owner had not observed that she was pregnant. The most common foetal cause was foetal malposition.

**Table 6:** Causes of dystocia

Cause	Number of cases	% of total
**Maternal**		
Partial uterine inertia	26	23.4
Complete uterine inertia	26	23.4
Uterine rupture	2	1.8
Iatrogenic uterine inertia	1	0.9
Uterine infection	1	0.9
**Total**	56	50.4
**Foetal**		
Malposition	13	11.7
Malformations	8	7.2
Kitten stuck	6	5.4
Dead kittens	5	4.5
Large foetus	3	2.7
**Total**	35	31.5
**Unclear**	20	18.0

## Discussion

Our study confirms that the mortality of kittens in feline dystocia cases is high, with a large proportion of deaths being stillbirths. The majority of stillborn kittens likely died *in utero* or in the birth canal before the initiation of treatment. This observation is supported by the finding that 29 out of 36 stillborn kittens showed no detectable heartbeat during ultrasound evaluation. Although the percentage of kittens that died before discharge increased with increasing interval between the first recorded sign of second-stage labour and admission, there was no significant effect of time in the second stage on mortality. However, the interval in the second stage was sometimes very uncertain. It is possible that some queens were in the second stage for a longer time than noticed by the owner. In addition, this information was not available for all cases. The relatively low number of cases in each interval may also have contributed to the low statistical power. The lack of association between time in the second stage, the inter-kitten interval, and kitten mortality has, however, also been observed in feline eutocia [7–9]. Kittens born before admission also had high mortality, indicating that the parturition process was disturbed already in many cases when kittens were born before veterinary assistance.

Among live-born kittens at the clinic, 20.0% died before being discharged (excluding four kittens that were euthanized at the owner’s request). It remains to be elucidated whether routine Apgar scoring might contribute to improved survival of live-born kittens [16].

Diagnosing dystocia in cats may be challenging. The majority of the cases had signs that were considered abnormal in both the dog and the cat, such as abnormal vaginal discharge, prolonged weak contractions, or strong non-productive contractions > 30 min [18]. A visibly stuck foetus is an obvious sign of dystocia that was present in 11.7% of the cases in this study. The mother being tired or distressed may be more difficult to assess but was recorded in almost 20% of the queens. The inter-kitten interval has not been reported to have a significant effect on the incidence of stillbirth [8, 10]. Although the birth of live kittens > 40 h after the previous birth has been reported, it seems to be unusual. An interval of 24–48 h between individual kittens was reported in 0.3% of births [3] reported that had an interval of 24–48 h. Similarly, the interval between the birth of the first and last kitten in the litter exceeded 6 h in only 2/58 cats [10]. The wide range of intervals complicates the diagnosis of dystocia in cats. Evaluation of foetal health with ultrasound may aid in distinguishing between abnormal and normal parturition in ambiguous cases. In a large study on feline reproductive performance, the incidence of stillbirth was 8.5%, and total mortality until weaning 16% [19]. This can be compared with the mortality of the kittens delivered at the animal hospital in this study, with a 29.7% stillbirth rate, and 44.1% mortality before discharge. The high incidence of stillbirths and neonatal mortality, along with the observed clinical signs, strongly suggests that most of the queens in this study experienced dystocia rather than a normal parturition mistakenly diagnosed as dystocia. The range of gestation length of 59–72 days in this study has previously been reported as normal in cats [2, 3, 8, 10, 20].

An overestimation of dead foetuses via ultrasound in some of the litters shows that sometimes it is difficult to obtain an accurate diagnosis in a patient who is in a stressful situation. A stressed queen that moves around will be more difficult to evaluate. The ultrasound expertise of veterinarians performing the exam may also vary, especially at hours when the ordinary diagnostic imaging staff may not be available. A greater number of stillborn foetuses than dead foetuses estimated via ultrasound in other litters is, on the other hand, not unexpected, as foetuses may die in the interval between the examination and delivery.

Surgical treatment was performed in 82.0% of all cases treated for dystocia. This percentage is much higher than the 56% reported in Swedish insurance data [5] or in a questionnaire study in the UK (85/154) [3] but similar to that reported in other studies on medical records or questionnaires (74.0-82.9%) [1, 13, 14]. In the present study, kitten mortality was significantly lower in patients treated with medication before CS than in those treated with CS without previous medical treatment attempts. This difference is likely caused by selection bias. It is unlikely that the longer time between admission and CS for queens that were first medically treated would favour kitten survival. Queens that are in good vigour without signs of foetal distress are more likely to be medically treated than queens that have more serious signs of dystocia. There is some controversy regarding the use of calcium for feline dystocia, as it has been reported that it may result in very strong contractions [21]. In this study, four queens delivered all remaining foetuses after treatment with calcium only, and 8/19 delivered all remaining kittens after treatment with calcium only or in combination with oxytocin. These findings indicate that calcium may also be a valuable medication for feline dystocia.

Medical treatment was initiated in only 27.0% of the cases. When it was initiated, the success rate was 36.7%, which is similar to previous reports on canine dystocia (30.3%) [22]. Medical treatment of dystocia has previously been reported to have a low success rate in cats, (29.0-29.9%) [6, 13], which was confirmed in this study. Calcium is necessary for efficient myometrial contractions, which is why the hypocalcaemia that was present in 8/90 queens may have contributed to dystocia in a minority of the cases. Only three of these patients were treated with calcium, one of which achieved complete success, but only after combination with oxytocin. Three other queens also responded with complete success to calcium infusion, although they were in the lower range of normocalcaemia. The benefit of calcium infusion in normocalcaemic individuals may also be at the cellular level [23]. Our data indicate that medical treatment may be a good option in patients with non-obstructive dystocia and with ≤ 3 unborn kittens.

Ovariohysterectomy was performed in 43 queens at the time of surgery. The proportion of OHE was significantly greater in queens that had undergone a CS in a previous pregnancy (83.3% vs. 29.6% of those that had not undergone a previous CS). According to Swedish animal welfare regulations, it is not allowed to breed from a queen that has given birth twice by CS, which may have contributed to a decision of OHE in queens undergoing their second CS [24]. Among the multiparous queens with known data, 28.6% had previously undergone CS. This finding indicates that continuing breeding from a queen that has suffered from dystocia is associated with an increased risk.

A majority of the queens were hyperglycaemic. Maternal hyperglycaemia, likely representing increased stress, was associated with significantly increased kitten mortality. This finding should be confirmed in more studies before any strong conclusions are drawn. Almost 22% of the queens had a low haematocrit. Maternal anaemia did not affect kitten mortality.

Ragdolls and British Short- or Longhair were the most common pedigree breeds in the present study. Although both breeds were overrepresented in insurance data on dystocia [5], they are also among the most popular breeds in Sweden. The Ragdoll breed had the most registrations in the largest Swedish cat registry during the entire study period (https://www.sverak.se/om-sverak/siffror-statistik/access 2024-03-27), whereas the British Shorthair was among the ten most popular breeds. There is no information about the geographical distribution of different cat breeds in Sweden. Therefore, it is not possible to draw conclusions about breed predisposition from medical records. The proportion of domestic cats with dystocia was greater in this study than in data evaluated from a Swedish insurance database (37.3% vs. 24.3%) [5] but was similar to that reported in an older study of Swedish clinical records (40%) [13]. Although the majority of Swedish cats are domestic [25], it is likely that a lower percentage of non-pedigree cats are used for planned breeding than pedigree cats. This group of cat owners is also more difficult to reach with questionnaires, as they are not organized in breed clubs. The greater proportion of domestic queens without information about mating date(s) could indicate that these litters tended not to be planned, in contrast to the pedigree litters. It is not possible to compare the risk of dystocia between pedigree and non-pedigree cats in studies using medical records or insurance databases, because of difficulties in collecting reliable data.

As described previously, the majority of feline dystocia cases seem to be of maternal origin, with uterine inertia being the most common cause [1, 6, 13, 14]. However, uterine inertia may sometimes be a clinical sign of another underlying cause [26]. A recent study revealed that pelvimetric measures were greater in British Shorthair queens with eutocia than in queens of the same breed with dystocia [27]. Pelvimetric measures are usually not performed during dystocia. An undiagnosed narrow birth canal could therefore be a cause of disturbed uterine contractions or kittens that are stuck in the birth canal. The presence of dead kittens may both be a cause of, or a consequence of, disturbed uterine contractions. Categorizing the cause of dystocia based on clinical signs and the reason for admission is likely to be very imprecise. Because of the retrospective nature of the study, we did not have access to data about the birth weights of the kittens. The evaluation of foetuses being large was based on, often subjective, clinical notes in the medical records. In a prospective study, it might be of interest to include data about relative foetal size in relation to the parturition process, as large foetuses may contribute to obstructive dystocia. The number of clinicians involved, a study period of seven years, and the acute nature of dystocia, probably contributed to a variability in completeness of records and choice of treatments.

## Conclusions

Our data confirmed that feline dystocia is associated with high kitten mortality but low mortality in queens. Most queens with dystocia are treated surgically, but medical treatment with calcium and/or oxytocin may be efficient in patients with non-obstructive dystocia and ≤ 3 foetuses remaining. Hypocalcaemia may contribute to dystocia in a minority of cases. Maternal hyperglycaemia increased the risk of kitten mortality before discharge. Diagnosing dystocia may be challenging in cats, as there is no clear association between the length of the parturition process and kitten mortality. Therefore, other signs of abnormal parturition, including findings at X-ray and ultrasound, are important for an accurate diagnosis.

## Data Availability

The datasets used and/or analysed during the current study are available from the corresponding author upon reasonable request.

## Declarations

### Prior publication

The data included in this article have previously been published in the Proceedings of the 1st European symposium on Animal Reproduction (ESAR), 21–23 September 2023, Nantes, France, and in the Proceedings of the 25th International EVSSAR Congress in Barcelona, Spain, 27–29 June 2024. Parts of the data have also been published as student theses in Swedish, with an English summary.
